# Epigenetic determinants of reproductive potential augment the predictive ability of the semen analysis

**DOI:** 10.1016/j.xfss.2023.09.001

**Published:** 2023-09-13

**Authors:** Ryan H. Miller, Elizabeth A. DeVilbiss, Kristin R. Brogaard, Carter R. Norton, Chad A. Pollard, Benjamin R. Emery, Kenneth I. Aston, James M. Hotaling, Tim G. Jenkins

**Affiliations:** a Inherent Biosciences, Salt Lake City, Utah; b Division of Population Health Research, Division of Intramural Research, Eunice Kennedy Shriver National Institute of Child Health and Human Development, Bethesda, Maryland; c Department of Cell Biology and Physiology, Brigham Young University, Provo, Utah; d Division of Urology, Department of Surgery, University of Utah School of Medicine, Salt Lake City, Utah

**Keywords:** Epigenetics, IUI, IVF, methylation, sperm

## Abstract

**Objective:**

To investigate the power of DNA methylation variability in sperm cells in assessing male fertility potential.

**Design:**

Retrospective cohort.

**Setting:**

Fertility care centers.

**Patients:**

Male patients seeking infertility treatment and fertile male sperm donors.

**Intervention:**

None.

**Main Outcome Measures:**

Sperm DNA methylation data from 43 fertile sperm donors were analyzed and compared with the data from 1344 men seeking fertility assessment or treatment. Methylation at gene promoters with the least variable methylation in fertile patients was used to create 3 categories of promoter dysregulation in the infertility treatment cohort: poor, average, and excellent sperm quality.

**Results:**

After controlling for female factors, there were significant differences in intrauterine insemination pregnancy and live birth outcomes between the poor and excellent groups across a cumulative average of 2–3 cycles: 19.4% vs. 51.7% (*P*=.008) and 19.4% vs. 44.8% (*P*=.03), respectively. Live birth outcomes from in vitro fertilization, primarily with intracytoplasmic sperm injection, were not found to be significantly different among any of the 3 groups.

**Conclusion:**

Methylation variability in a panel of 1233 gene promoters could augment the predictive ability of semen analysis and be a reliable biomarker for assessing intrauterine insemination outcomes. In vitro fertilization with intracytoplasmic sperm injection appears to overcome high levels of epigenetic instability in sperm.

A systems biology approach is required to fully understand the complexity of spermatogenesis, fertilization, and embryo development (1, 2). Investment in research over the last 2 decades has revealed many multifactorial relationships among sperm DNA, RNA, microRNAs, DNA methylation, chromatin, and the proteome (3–6) in each step of conception and embryo development. Despite these advancements, male infertility is still assessed through basic semen analysis which consists of a visual examination for sperm quantity, shape, and motility.

The semen analysis has changed very little over past decades other than minor modifications in the assessment of morphology made by the World Health Organization in 2021 (7). Although numerous studies have identified semen analysis parameters as important benchmarks for evaluating reproductive health, its power to predict fertility outcomes remains limited (8–10). The introduction of DNA fragmentation testing has provided additional insights into the molecular function of sperm by assessing the structural integrity of sperm DNA. However, because of the lack of correlation with fertility potential, current guidelines and research suggest that DNA fragmentation should not be tested in the initial assessment of male infertility, except for in cases of recurrent pregnancy loss. Thus, the semen analysis remains the primary tool for initial male fertility assessment (7).

Implementation of the semen analysis as the primary, and in most cases, the only, assessment of sperm health leads to an incomplete understanding for couples seeking fertility care. Consequently, this incomplete understanding often results in men not being identified as subfertile, leading to unnecessary procedures, a longer time-to-pregnancy, and an increased burden on the female partner (2). Advances in the assessment of male fertility are needed for more comprehensive diagnostics and approaches for the identification and treatment of male infertility.

Epigenetic analysis of sperm DNA has emerged in recent years as a potential tool for a more comprehensive assessment of male fertility potential (11–15). “Epigenetics” refers to the heritable regulation of gene expression independent of changes to the DNA sequence itself. Specifically, the analysis presented here assesses DNA methylation modifications that occur at cytosine-phosphate guanine dinucleotides in DNA. Because DNA methylation helps control gene expression, the maintenance of proper DNA methylation is crucial for healthy sperm function (16). The objective of this study is to better understand the epigenetic determinants of sperm quality and assess them as a new diagnostic for determining male fertility potential.

Utilizing data from a multi-site National Institutes of Health clinical trial (17), we employed a novel method for the analysis of aberrant DNA methylation and global quantification of genes crucial for sperm function (18). After analysis of 1344 semen samples, we introduced the discovery of an epigenetic (DNA methylation) profile that shows promise in assessing sperm quality, termed Epigenetic Sperm Quality Test (SpermQT) for this study. Taken together with semen analysis, SpermQT could expand the clinical assessment of male fertility potential.

## MATERIALS AND METHODS

### Data Procurement

Sperm DNA methylation data (Infinium MethylationEPIC Array) from fertile sperm donors were obtained from Miller et al. (18). Additionally, sperm DNA methylation data from a clinical multi-site National Institutes of Health study of men experiencing infertility were used from previously published data from Jenkins et al. (17). The trial was approved by the Institutional Review Boards at all study centers and the data coordinating center. Written informed consent was obtained from all participants, and a Data and Safety Monitoring Board provided external oversight.

### Patient Details

Analysis was completed on 1344 de-identified patient sperm DNA methylation data and clinical outcomes previously published in Jenkins et al. (17). Outcomes included both live birth and pregnancy data, where pregnancy was determined by either ultrasound or biochemical (human chorionic gonadotropin) assessment. The clinical information of patients was described in detail in the previous publication. The population of couples undergoing intrauterine insemination (IUI) completed a cumulative average of 2–3 cycles during the study. For couples undergoing in vitro fertilization (IVF), a cumulative average of 1–2 embryos were transferred per couple and 76% of fertilization occurred via IVF with intracytoplasmic sperm injection (ICSI) (19). Additionally, when controlling for female factors, women <35 years old with no prior diagnosis of polycystic ovary syndrome, endometriosis, fibroids, blocked tubes, or diminished ovarian reserves were included.

### Data Pre-Processing

The sperm DNA methylation data were preprocessed as described in Miller et al. (18) with minor modifications as detailed in Supplemental File 1 (available online). We also removed any sperm samples from analysis that did not have a mean methylation value less than 0.24 of all the cytosine-phosphate guanine dinucleotide beta values in the differentially methylated region of DLK1 as described by Jenkins et al. (20) (chr14:101,191,893–101,192,913, GRCh37). Jenkins et al. (20) showed the methylation states of the probes in this region are a good discriminator between sperm and somatic cells. This procedure ensured analyses were performed only on samples containing sperm DNA methylation and not contaminating somatic cell DNA methylation.

### Statistical Analyses

Gene promoters with the least variable methylation values (n = 1233) in sperm from fertile sperm donors (n = 43) and the corresponding gene promoter variability cutoffs were selected as described in Miller et al. (18). These promoters and corresponding cutoffs were then used to perform sperm methylation variability analyses on the sperm methylation data from men experiencing infertility (n = 1344). We observed the promoter methylation variability within selected promoters and identified the number of promoters falling outside the prescribed gene methylation promoter cutoffs (i.e., “dysregulated promoters”). These analyses were performed as outlined in Miller et al. (18) with a minor modification noted in Supplemental File 1. In addition, an ontology analysis was performed on these 1233 promoter regions using the web application implementation of the GREAT algorithm (https://great.stanford.edu) (21) as shown in Supplemental Figure 1 (available online).

We then established thresholds for the number of dysregulated promoters for samples with “Excellent” (≤ 3 dysregulated promoters), “Average” (between 4 and 21 dysregulated promoters), and “Poor” sperm quality (≥22 dysregulated promoters). Two-sided t-tests were subsequently performed on the pregnancy and live birth outcomes of these couples, categorized by sperm quality and the type of infertility treatment received.

A permutation analysis (n = 10,000) was performed by shuffling the live birth results of couples receiving IUI treatment, and the live birth rates of couples in the Excellent sperm quality category was compared with those in the Poor sperm quality category (Supplemental Figure 2, available online).

## RESULTS

### Demographic and Methylome-Wide Analysis of Promoter Dysregulation from 1344 Men Seeking Fertility Care

Full semen parameters and male demographic information can be found in Supplemental Table 1 (available online). Of the 1344 men analyzed for this study, 12.0% had a sperm concentration of less than 15 million/mL, 14.3% had a total motile count (TMC) less than 20 million, and 65.5% had morphology results greater than or equal to 4.0%. An overview of the female partner demographics can be found in Supplemental Table 2 (available online). In an additional analysis of IUI-treated fertility outcomes, 21.1% of men were removed from the study because their partners had known female infertility factors.

**FIGURE 1:**
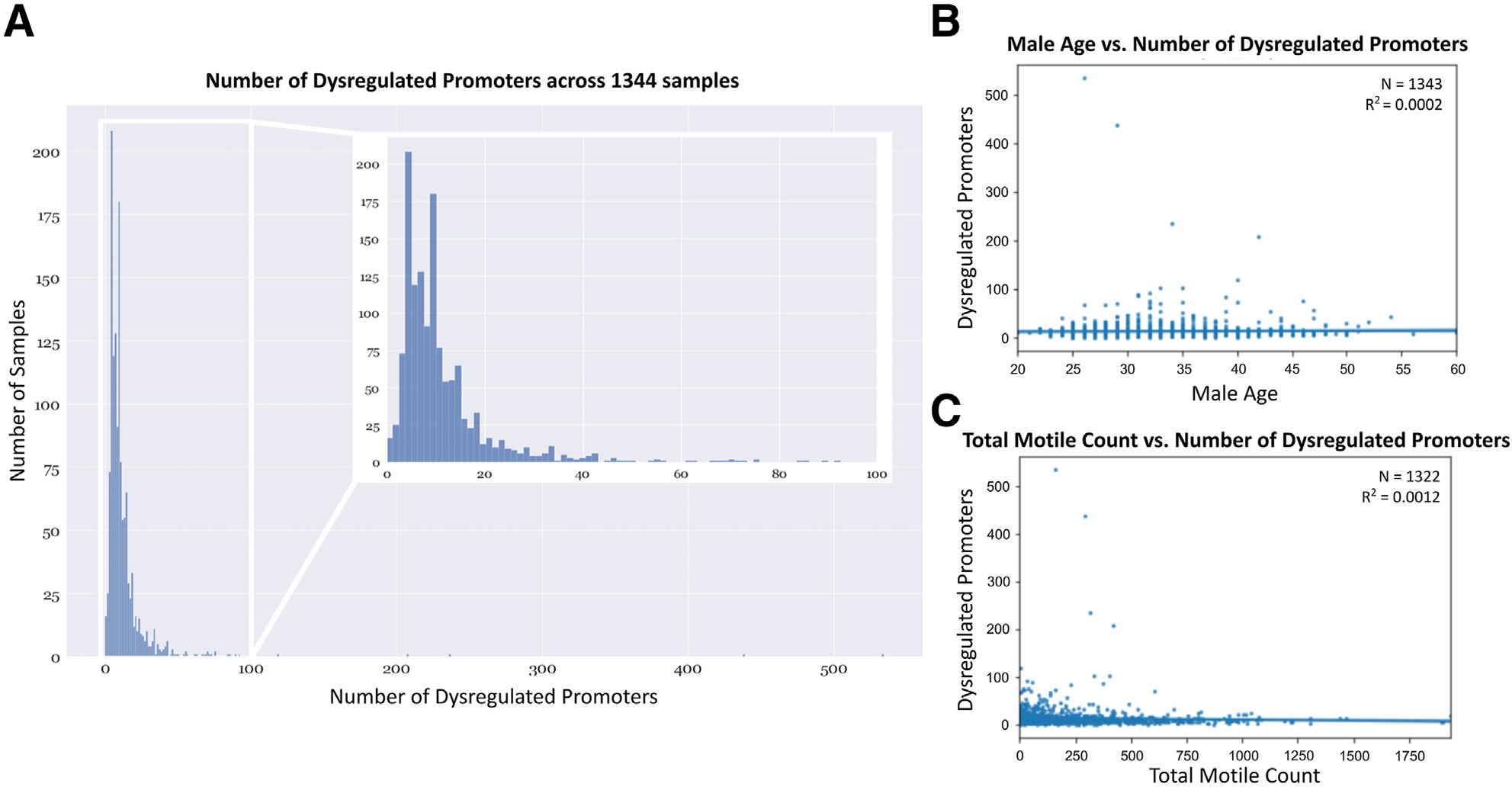
Number of dysregulated promoters does not correlate with participant age and sperm motility. **(A)** Distribution of dysregulated promoters across sample cohorts. **(B)** No significant relationship exists between participant age and number of dysregulated promoters at R^2^ = 0.0002. **(C)** No significant relationship exists between total motile count of sperm and number of dysregulated promoters at R^2^ = 0.0012.

We performed a gene promoter methylation variability analysis on sperm samples from 1344 men seeking infertility care to quantify how many genes had dysregulated promoters. Thresholds for these signatures of irregular methylation were set on the basis of fertile controls. Distribution of dysregulated promoters among the infertile men displayed an average of 12.7 dysregulated promoters and a median of 9.0 dysregulated promoters (Fig. 1A). We performed regression analyses between the number of dysregulated promoters and several factors such as male BMI, age, TMC, concentration, and morphology of sperm, and found no meaningful relationships to the number of dysregulated promoters (Fig. 1B and C, Supplemental Figure 3, available online).

### Increasing Prevalence of Dysregulated Promoters is Associated with Lower Pregnancy and Live Birth Outcomes in IUI Procedures

Previous data in other disease types had shown that increased promoter dysregulation is associated with pathologic phenotypes (18). We sought to understand the relationship between the number of dysregulated promoters and clinical outcomes for different fertility treatments while controlling for female infertility factors. To do this, the top and bottom 10^th^ percentile of dysregulated promoters were identified to the nearest integer. The top 10th percentile included men with ≥22 dysregulated promoters (n = 140) and was designated as the “Poor” sperm quality group. The bottom 10th percentile of dysregulated promoters included men with ≤3 dysregulated promoters (n = 114) and was designated as the “Excellent” sperm quality group. All remaining men with >3 and <22 dysregulated promoters (n = 1090) were designated as the “Normal” sperm quality group. Supplemental Table 1 contains the semen parameters and demographics associated with each group. When creating these 3 distinct groups, we identified a statistically significant enrichment of men with low TMC in the Poor group compared with the Excellent group.

**FIGURE 2:**
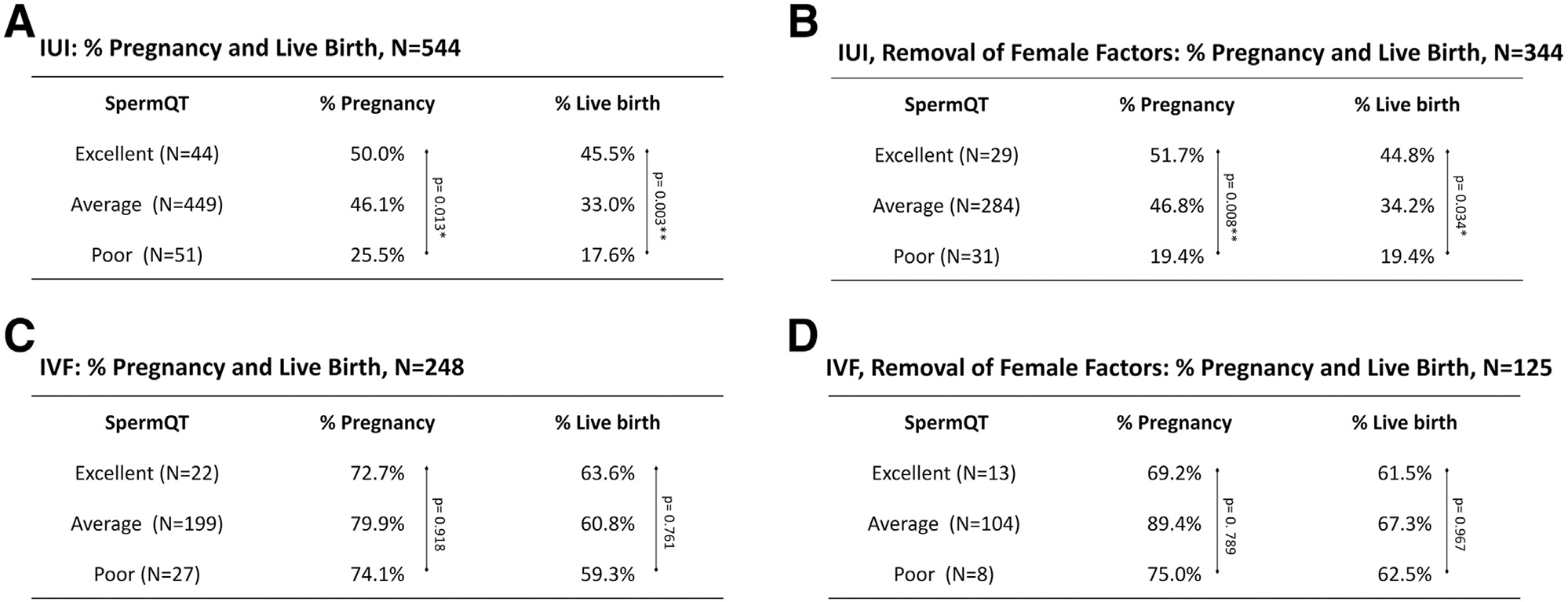
High prevalence of dysregulated promoters is associated with low fertility outcomes among IUI-treated couples. **(A)** Proportion of pregnancies and live births among IUI couples with Excellent sperm quality were significantly greater than couples with Poor sperm quality, both before and **(B)** after excluding couples affected by female infertility factors. **(C)** Proportion of pregnancies and live births were not significantly different for IVF couples (primarily with ICSI), neither before nor **(D)** after excluding couples affected by female infertility factors. ICSI = intracytoplasmic sperm injection; IVF = in vitro fertilization; IUI = intrauterine insemination.

Analysis of the percentage of live births and pregnancies of couples undergoing IUI (n = 544) showed a statistically significant difference between the Excellent and Poor sperm quality groups, as well as between the Average and Poor sperm quality groups (Fig. 2A). Similar pregnancy and live birth results were seen for couples whose female partners had no female infertility factors (n = 344) (Fig. 2B), indicating a relationship between sperm DNA methylation promoter dysregulation and fertility potential. A permutation analysis was completed to determine if the differences seen in live birth rates could be due to random chance. We found the real difference in live birth rates to be in the 99.5 percentile of permutations, indicating a very low probability that these results are due to chance (Supplemental Figure 2).

When completing the same analysis for men undergoing IVF (primarily with ICSI), we saw no statistical difference between any of the sperm quality groups (Excellent, Average, or Poor), with or without controlling for female factors (Figs. 2C and D). These data together show that the analysis of accumulated dysregulated promoters, termed Epigenetic SpermQT, appears to identify men with lower fertility potential for IUI procedures. Additionally, IVF appears to overcome sperm quality issues identified with SpermQT.

### The Number of Dysregulated Promoters Combined with TMC Is More Predictive of Pregnancy and Live Births than TMC Alone

**FIGURE 3:**
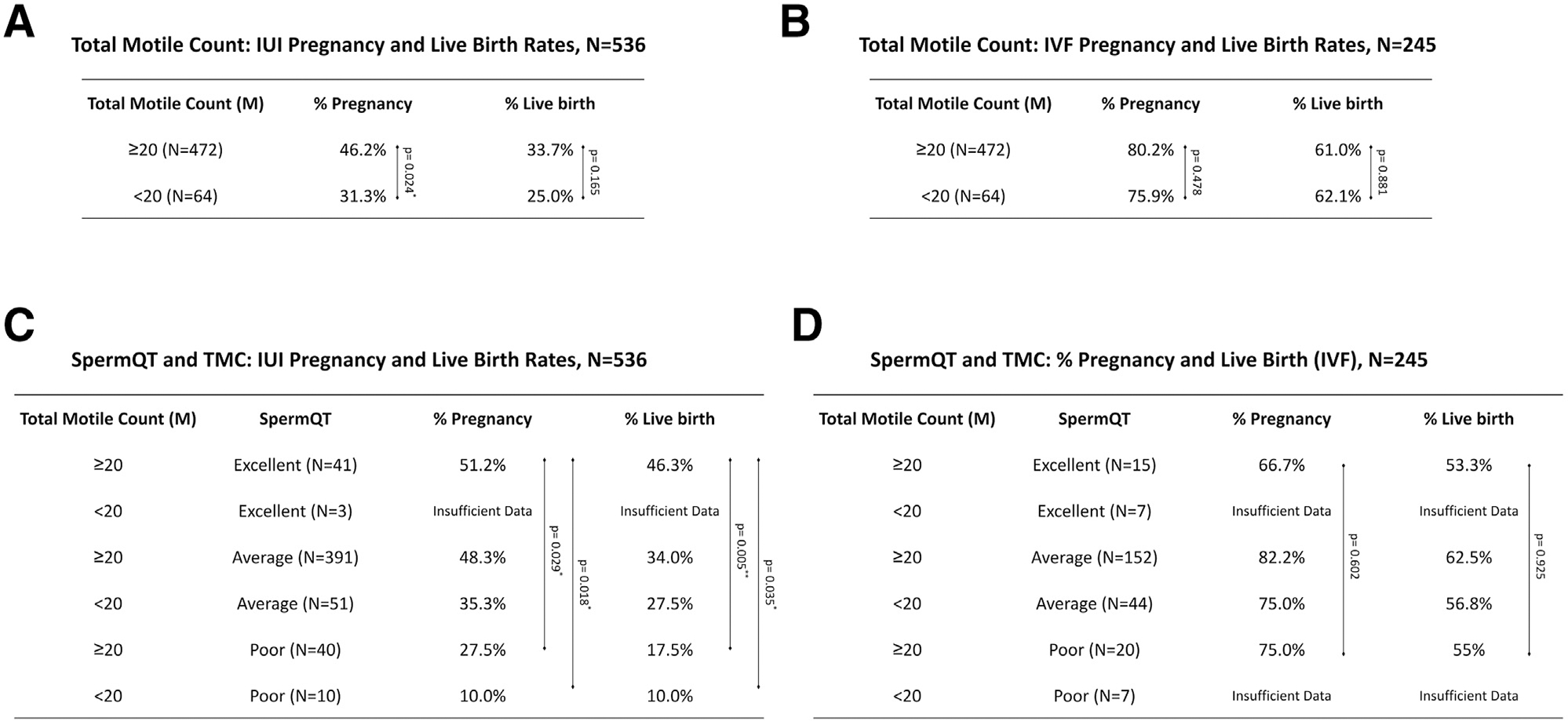
Synergistic effect of combining TMC with SpermQT metrics for assessing IUI outcomes. **(A)** Pregnancy and live birth results of couples with TMC of ≥20 M or TMC <20 M receiving IUI. **(B)** Pregnancy and live birth results of couples with TMC of ≥20 M or TMC <20 M receiving IVF (primarily with ICSI). **(C)** Pregnancy and live birth results of couples receiving IUI, stratified by TMC and SpermQT result. **(D)** Pregnancy and live birth results of couples receiving IVF (primarily with ICSI), stratified by TMC and SpermQT result. ICSI = intracytoplasmic sperm injection; IVF = in vitro fertilization; IUI = intrauterine insemination; TMC = total motile count.

Even with enrichment of low TMC in the Poor sperm quality group (Supplemental Table 1), 77.8% of men with a Poor SpermQT result had a TMC ≥20 M and 75.6% had both a TMC ≥20 M and a sperm concentration ≥15 M/mL, suggesting that SpermQT identifies a new subset of men with low fertility potential that would have been missed by semen analysis alone. Interestingly, in these data, TMC alone is not statistically predictive of live birth rates in individuals undergoing IUI (Fig. 3A). However, when combined with SpermQT, the integration of both assessments provides a statistically significant prediction of live births from IUI (Fig. 3C). Similar to SpermQT alone, the combination of TMC with SpermQT is not predictive of outcomes of IVF (primarily with ICSI) (Fig. 3D). With both tests identifying a unique subset of subfertile men, the combination of SpermQT with TMC could provide a more comprehensive assessment of male fertility, identifying previously undiagnosed men as subfertile and helping physicians guide their treatment recommendations more accurately.

### SpermQT Analyzes an Accumulation of Dysregulation in Multiple Biological Pathways

**FIGURE 4:**
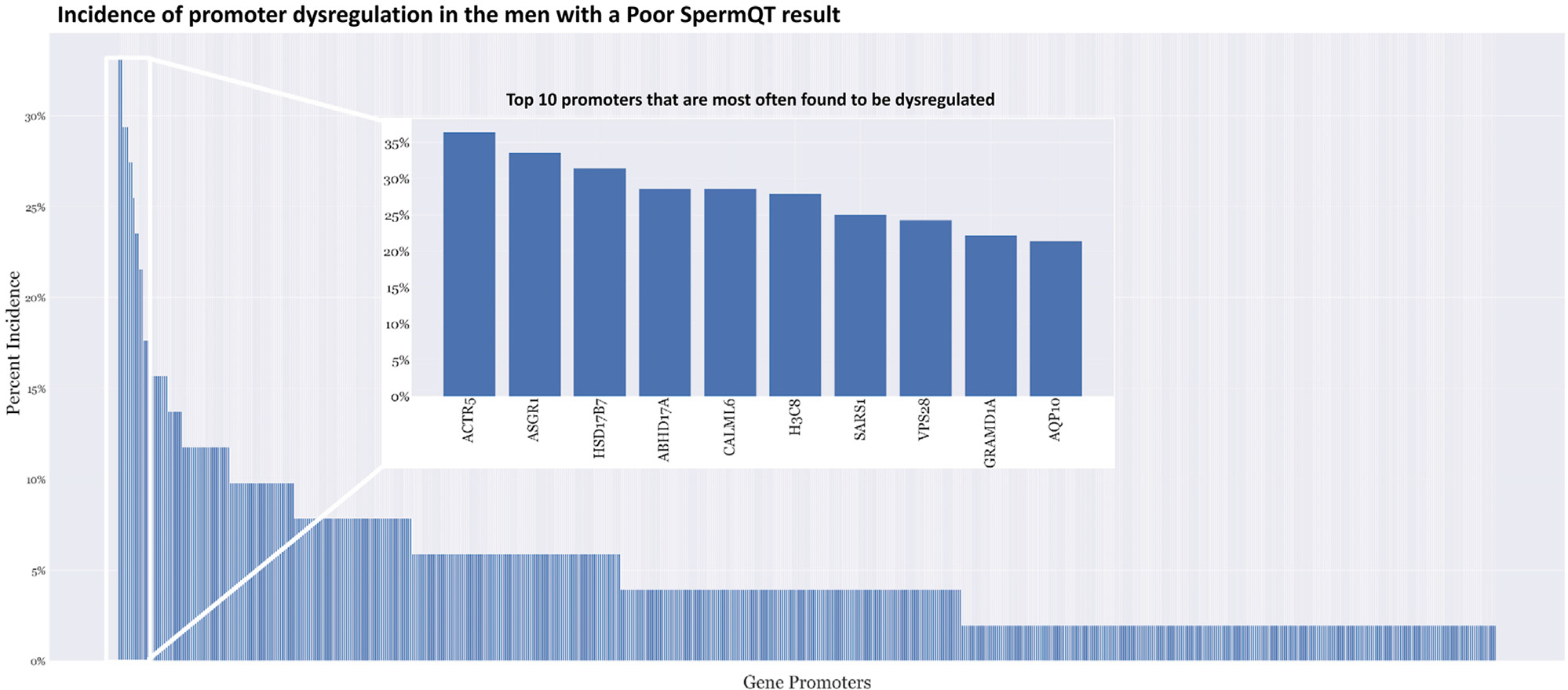
Promoter dysregulation is highly heterogeneous in men with poor SpermQT results. Incidence of promoter dysregulation among men with poor SpermQT results does not appear to be highly conserved.

Analysis of the dysregulated promoters in 1233 target gene promoters across all samples with a Poor SpermQT score revealed a broad distribution of irregular methylation (Fig. 4). This reflects the biological complexity and heterogeneity between male infertility patients. Ten gene promoters were epigenetically dysregulated in more than 20% of samples (Fig. 4 inset) with 3 dysregulated genes (ACTR5, ASGR1, and HSD17B7) present in more than 30% of samples (36.2%, 33.3%, and 31.2%, respectively). ACTR5 is an Actin Repair Protein known for UV-damage repair and double-strand break repair and has been previously identified to be highly expressed in the testis (22). ASGR1 is a protein subunit of the asialoglycoprotein receptor largely known for glycoprotein homeostasis in the liver. However, ASGR1 has also been identified as enriched in early and late-stage spermatids because of glycoproteins’ essential role in sperm development and function (21). HSD17B7 is an enzyme involved in estrogen and androgen metabolism as well as cholesterol biosynthesis. Deletion of HSD17B7 has been shown to cause reduced testosterone production and early fetal death in mice (23, 24). Additional analysis of the distribution of dysregulated promoters can be found in Supplemental Figure 4 (available online). We also performed an ontology analysis (21) on these 1233 promoter regions to gain more insight into the possible pathways and mechanisms contributing to this phenotype of subfertility. Analysis showed 4 terms were enriched in the GO Molecular Function ontology: peptidase regulator activity, peptidase inhibitor activity, endopeptidase regulator activity, and endopeptidase inhibitor activity (Supplemental Figure 1). Interestingly, it has been hypothesized for years that these enzymes could play a role in the physiology of sperm (25), with more recent work in mice and humans supporting this hypothesis (26–30).

## DISCUSSION

This study was grounded in the concept that there are multiple biological pathways that may lead to decreased fertility potential, and that these biological pathways likely differ among infertile men. Here, we have defined a threshold for epigenetic stability that serves as an indicator of “healthy” sperm. Once a man’s sperm crosses this threshold, there emerges a phenotype of lower fertility potential.

We have shown the development of a DNA methylation assessment in sperm that has a statistically significant association with pregnancy and live birth percentages of couples undergoing IUI treatment. SpermQT’s ability to identify a subset of men that are largely missed by the current standard of care could allow for a more comprehensive assessment of male fertility by healthcare providers. For example, men with Poor sperm quality results may be advised to forgo IUI treatment in favor of IVF with ICSI, saving the patient time and expenses.

Male infertility is a complex disorder that will require a combination of assessments for physicians to understand it more effectively. The primarily visual and superficial aspects of the current standard of care (basic semen analysis) are useful but fall short of a comprehensive diagnostic for male infertility. When combined with the initial semen analysis (particularly TMC), SpermQT metrics may provide more guidance and help set expectations for couples seeking infertility care. To ensure these results are conserved across population types, we call for additional independent, prospective studies to further validate the application of this potential diagnostic test. Because this study was conducted with retrospective data, future studies on prospective data are needed for continued validation of this new sperm assessment. One prospective clinical trial using this biomarker is already in progress, furthering this important research (https://clinicaltrials.gov/study/NCT05966883). Additionally, future analysis with sperm RNA and protein analysis is needed to validate differences in gene expression between men with Excellent (or Normal) and Poor SpermQT results. Future work into the relationship of peptidase/endopeptidase regulator activity as well as peptidase/endopeptidase inhibitor activity and male infertility could also prove very fruitful as indicated by the ontology analysis of these 1233 promoters. We believe future studies coupling epigenetic and gene expression patterns as well as chromatin structure will be important to give more insights into the epigenetic underpinnings and overall mechanism of this biomarker. Future knowledge of the mechanism(s) identified by this potential biomarker could also be invaluable in developing future infertility therapeutics.

We anticipate that the clinical analysis of sperm DNA methylation will become increasingly relevant, as it has been shown that sperm DNA methylation can change in response to environmental exposures, diet, lifestyle, and medications (13, 31–33). Future research is in progress on the effects of these types of changes in decreasing the number of dysregulated promoters and improving fertility outcomes.

## CONCLUSION

Our retrospective analysis supports 2 key findings: First, infertile men in our sample population displayed greater numbers of dysregulated promoters than healthy sperm donors. Second, analysis of these dysregulated promoters can assess IUI treatment outcomes. The lack of a similar correlation for IVF treatment outcomes is encouraging because it suggests that there is no loss of fitness because of dysregulated promoters with the assistance of IVF (primarily with ICSI). Moreover, when paired with the traditional semen analysis, SpermQT could direct clinicians and couples toward the most effective treatment plan. Building on this research will not only improve clinical outcomes, but provide longoverdue guidance for those seeking infertility care.

## Supplementary Material

MMC1

Supp.table 2

Supp.Fig4

Supp.table 1

Supp.Fig2

Supp.Fig1

Supp.Fig3
