# Supplementary material for: Epigenetic determinants of reproductive potential augment the predictive ability of the semen analysis
Source: F S Sci. Author manuscript; Available in PMC 2024 Feb 5. (PMC10843460; doi:10.1016/j.xfss.2023.09.001)
Supplement: MMC1 [file NIHMS1938052-supplement-MMC1.pdf]

Data preprocessing modifications

The following are the data preprocessing steps for sperm DNA methylation that were modified from Miller et al:

1. No normalization was used to produce the beta and m-values from the methylation array data.
2. We removed all infinity and negative infinity beta and m-values that resulted from not normalizing the methylation data.
3. We removed all beta and m-values from analysis whose corresponding detection p-values were less than 5e-10.

Variability analysis modifications

With the variability methylation value of a gene promoter defined by Miller et al as:

$$\sigma = \sqrt{\frac{\sum |x_1 - \mu|^2}{N}}$$

where x_1 = m-value of a given Illumina EPIC array probe in a given gene promoter, μ = mean of the EPIC array probes m-values in that given promoter, we modified the equation to calculate the methylation variability cutoff threshold for a given promoter as:

$$\theta = 1.1(\frac{\sum \sigma_1}{N} + 3\sqrt{\frac{\sum |\sigma_1 - \mu|^2}{N}})$$

where σ_1 = promoter methylation variability value of a sample within a given cohort at a given promoter and μ = mean of the given promoter methylation m-values in a given cohort.
