# Supplementary figures and images for: Epigenetic determinants of reproductive potential augment the predictive ability of the semen analysis
Source: F S Sci. Author manuscript; Available in PMC 2024 Feb 5. (PMC10843460; doi:10.1016/j.xfss.2023.09.001)

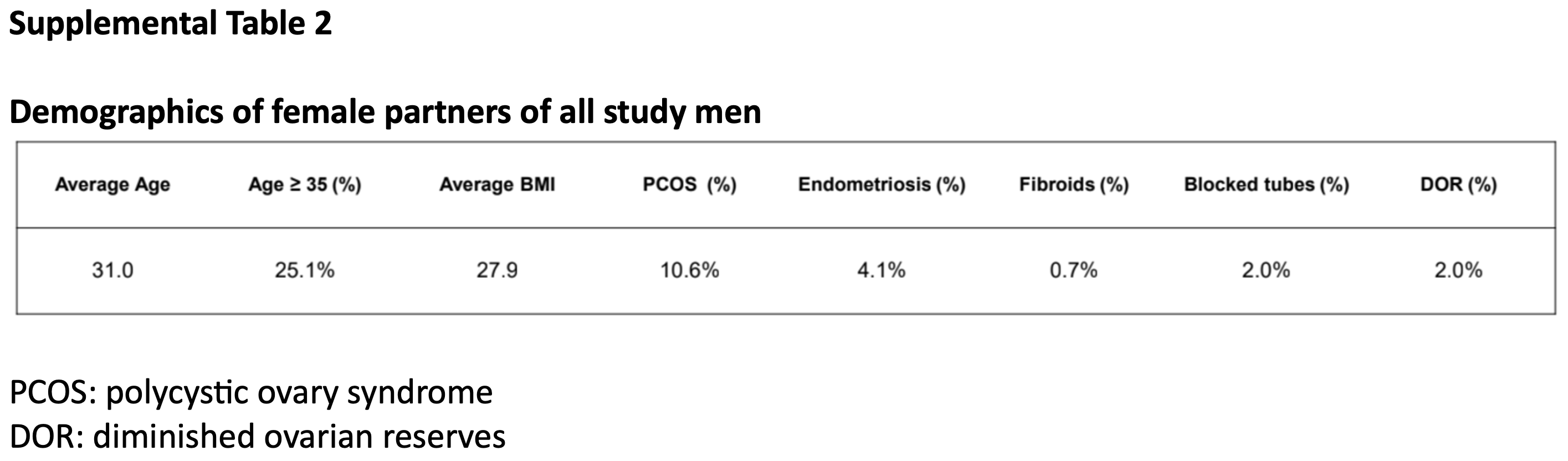

Supplement: Supp.table 2 [file NIHMS1938052-supplement-Supp_table_2.png]

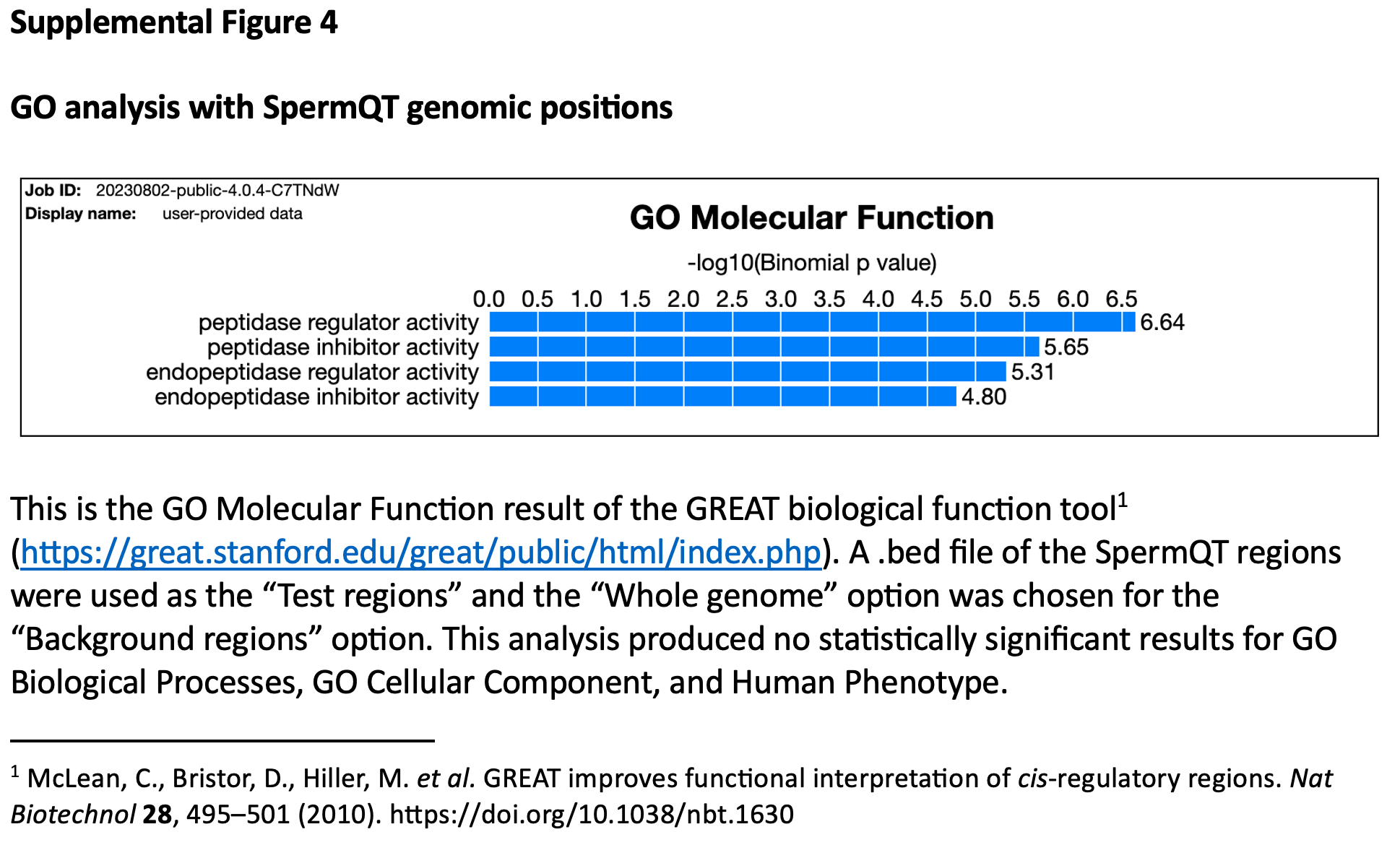

Supplement: Supp.Fig4 [file NIHMS1938052-supplement-Supp_Fig4.png]

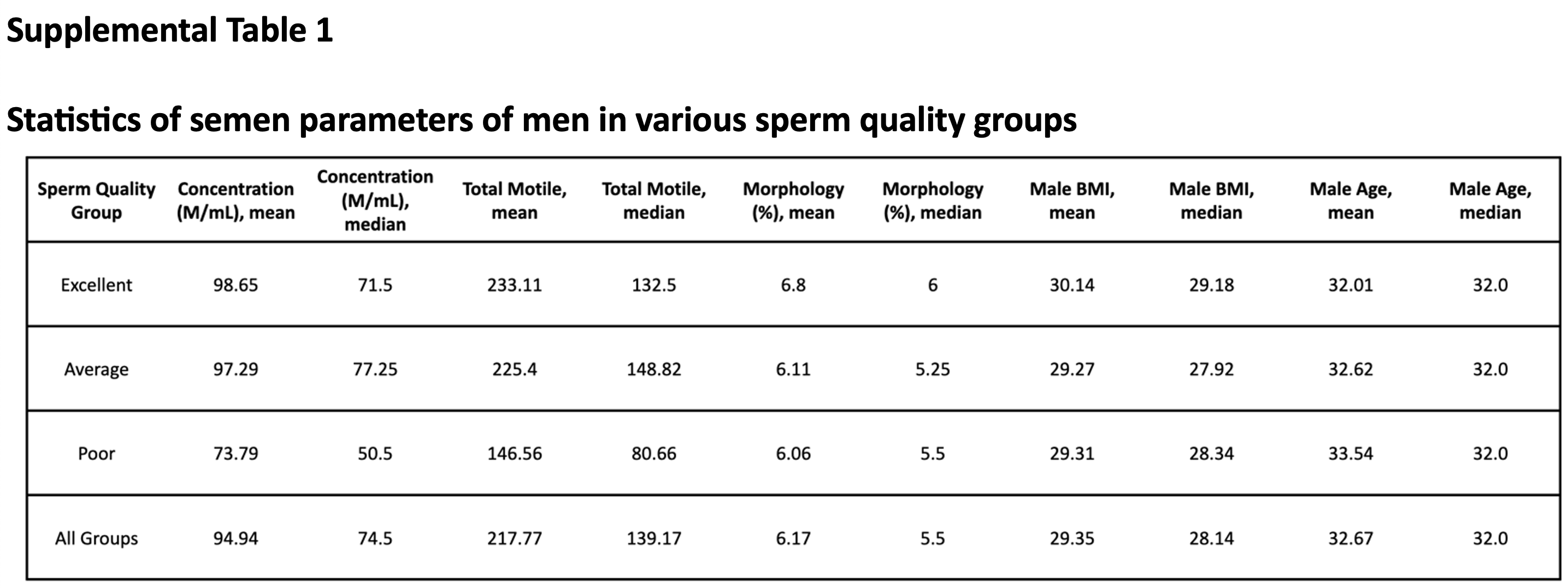

Supplement: Supp.table 1 [file NIHMS1938052-supplement-Supp_table_1.png]

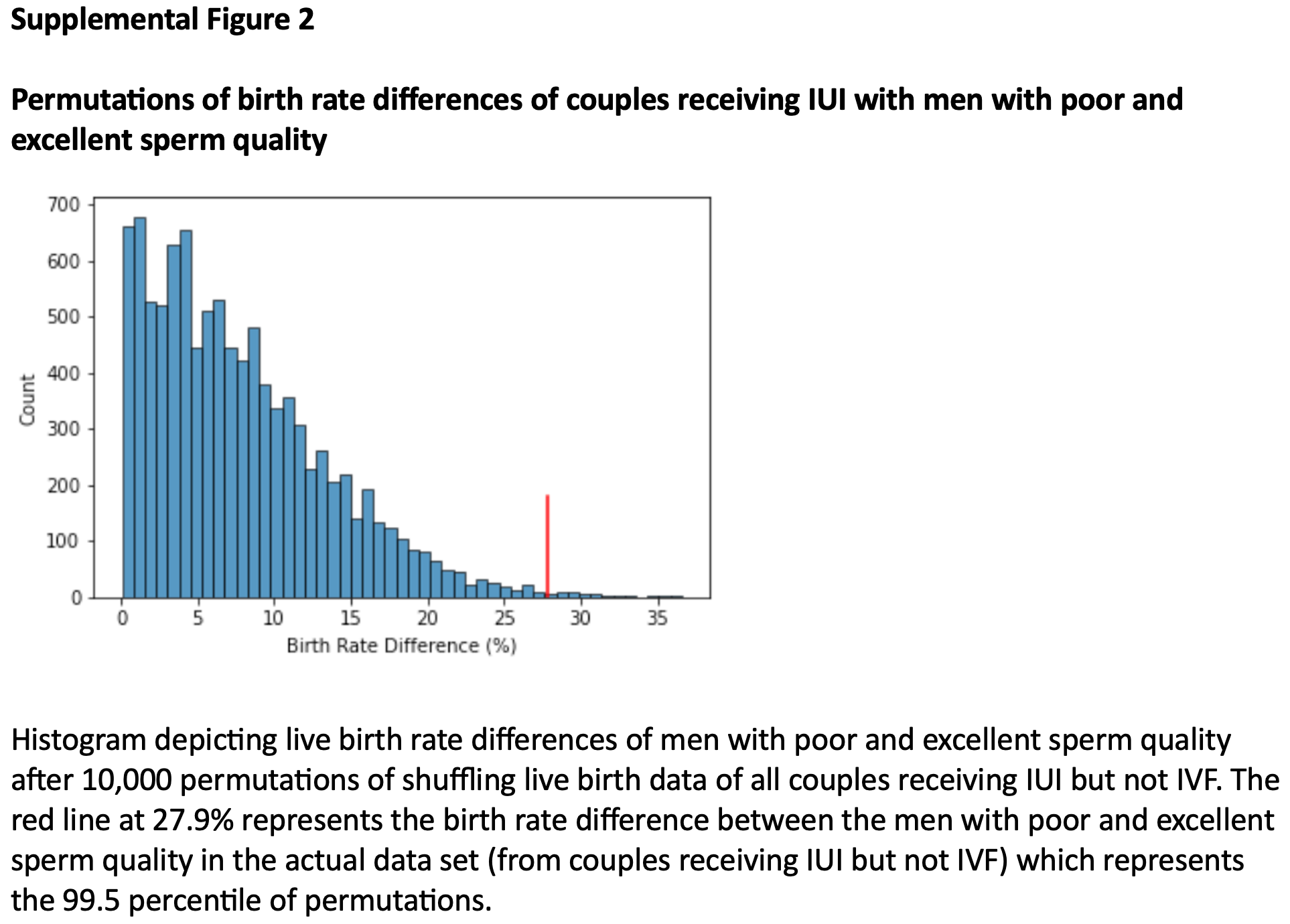

Supplement: Supp.Fig2 [file NIHMS1938052-supplement-Supp_Fig2.png]

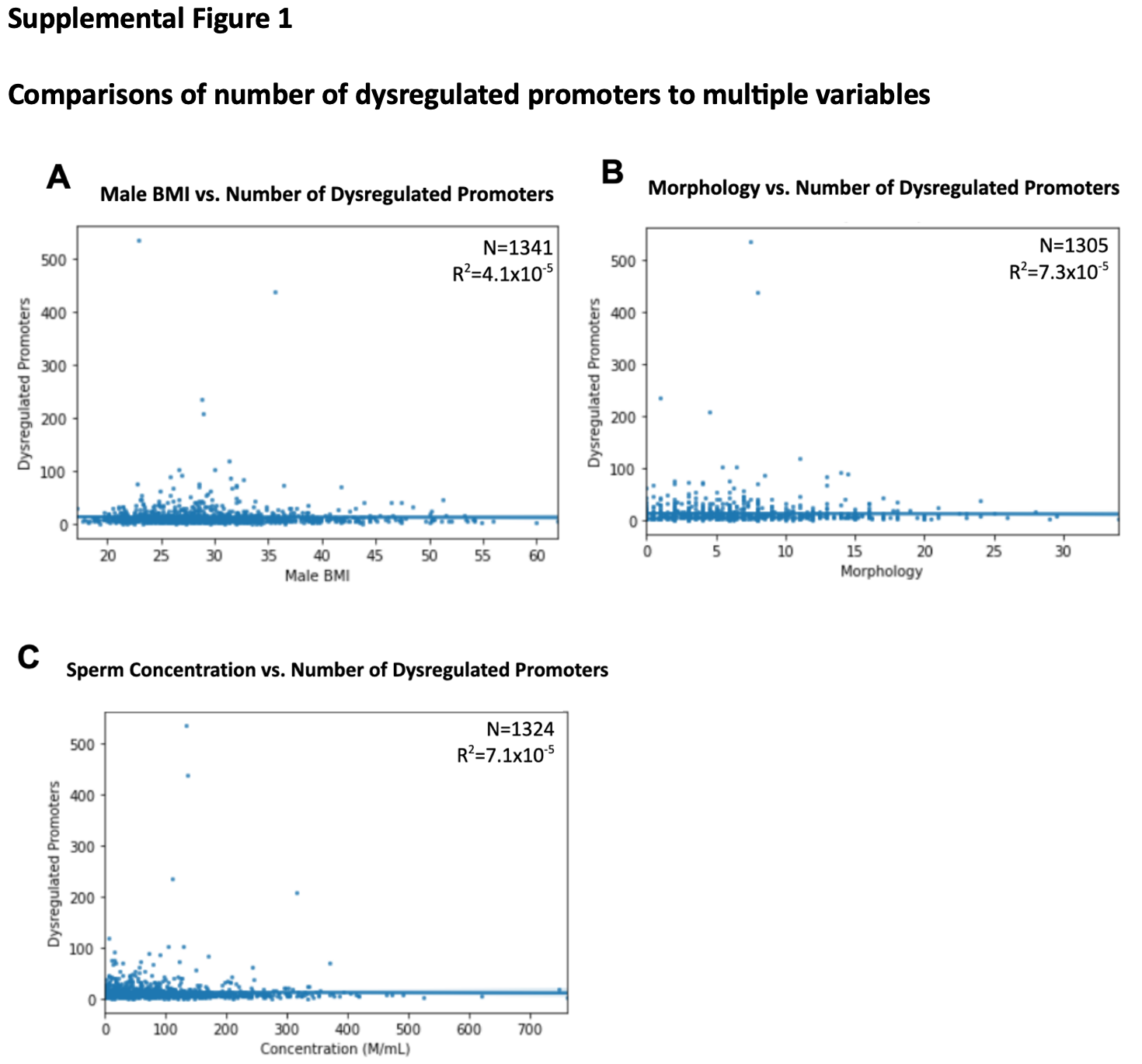

Supplement: Supp.Fig1 [file NIHMS1938052-supplement-Supp_Fig1.png]

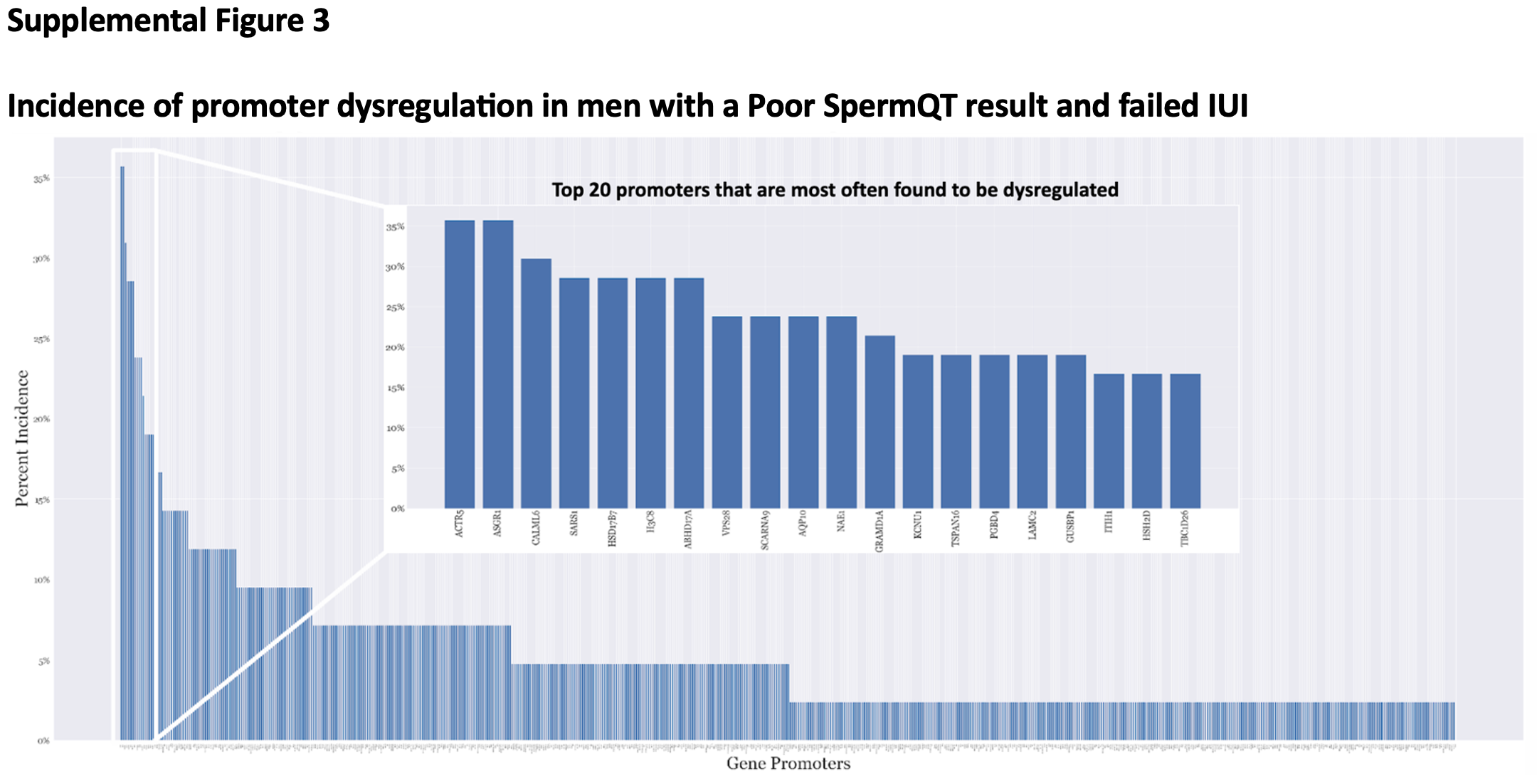

Supplement: Supp.Fig3 [file NIHMS1938052-supplement-Supp_Fig3.png]
